# Monthly Summary

**Published:** 1888-12

**Authors:** 


### Monthly Summary.

Death by Electricity: the New Method of Executing Criminals.—The law requiring electrical executions of death-sentences will go into effect on January 1st. The experiments on dogs made last summer by Harold P. Brown, an electrical engineer, were criticised because the weight of the animals killed was less than that of a man, and it was supposed that more current would be required to kill a human being on that account. On Wednesday afternoon Mr. Brown had an opportunity to make a demonstration before Mr. Elbridge T. Gerry, the author of the new law, and the committee appointed by the Medico-legal Society to report on the best means of putting the law into effect. It was sought to find out the amount and character of current that would be required to make death certain and instantaneous. The experiments were made in Mr. Edison's laboratory in Orange. The first victim was a calf weighing 124½ pounds. The hair was cut on the forehead and on the spine behind the forelegs, and sponge-covered plates, moistened with a solution of sulphate of zinc, were fastened in place. The resistance of the animal was 3.200 ohms. An alternating current of 700 volts was applied for thirty seconds. The animal, however, was killed instantly. It was at once dissected by Drs. Ingram and Bleyer, but the brain, heart, and lungs, were found to be in a normal condition, and the meat was pronounced fit for food. One metal plate carrying the current touched the hair of the forehead and slightly burned it, but otherwise there were no external indications of injury. A second calf weighed 145 pounds and had a resistance of 1,300 ohms. The deadly alternating current at 700-volts pressure was applied for five seconds, and produced instant death. To settle permanently the weight-question, a horse weighing 1.230 pounds was next killed by passing the alternating current at 700 volts from one foreleg to the other. The resistance of this animal was 11,000 ohms. Mr. Brown was assisted by Mr. A. E. Kennelly, and there were present Mr. Thomas A. Edison, Professor R. Ogden Doremus, Professor Charles A. Doremus, Dr Frederick Peterson, Dr. Frank H. Ingram, Mr. Elbridge T. Gerry, Mr. Galvin, Dr. J. M. Bleyer, H. Bourgouon, and Mr. John Murray Mitchell. The experiments are said to have proved the alternating current to be the most deadly force known to science.—*Medical Record.*

